# Self-assembly of surface layer protein SbsC from *Geobacillus stearothermophilus* and common principles of Bacilli S-layers

**DOI:** 10.1016/j.jbc.2026.113461

**Published:** 2026-08-24

**Authors:** Christoph Grininger, Lavinia Gambelli, Anđela Đordić, Markus Eder, Christoph Buhlheller, Theo Sagmeister, Gustav Oberdorfer, Janet Vonck, Uwe B. Sleytr, Walter Keller, Bertram Daum, Tea Pavkov-Keller

**Affiliations:** 1 Institute of Molecular Biosciences, NAWI Graz, University of Graz, Graz, Austria; 2 Living Systems Institute, University of Exeter, Exeter, United Kingdom; 3 Library Services Research Support, University of Bristol, Bristol, United Kingdom; 4 Institute of Biochemistry, University of Technology, Graz, Austria; 5 BioTechMed-Graz, Graz, Austria; 6 Department of Structural Biology, Max Planck Institute of Biophysics, Frankfurt am Main, Germany; 7 Institute of Synthetic Bioarchitectures, University of Natural Resources and Life Sciences Vienna, Vienna, Austria; 8 BioHealth, Field of Excellence BioHealth – University of Graz, Graz, Austria

**Keywords:** surface layer protein, S-layer, SbsC, gram positive bacteria, self-assembly

## Abstract

S-layers are (glyco)protein coats displayed on the external surface of many bacterial and archaeal species. By combining X-ray crystallography, electron cryo-tomography and SymProFold predictions, we propose the structure of the self-assembled S-layer protein (SLP) SbsC from the Gram-positive bacterium *Geobacillus stearothermophilus* ATCC 12980. SbsC consists of nine domains arranged like beads on a string. Domains 2 through 7 form a closed ring-like conformation, while the secondary cell wall polymer binding domain 1 and the auxiliary domains 8 and 9 protrude from the ring. Within the assembled lattice, the molecules adopt *p*1 symmetry and conform to the curvature of the bacterial surface, creating a sub-S-layer space, an enclosed space between the peptidoglycan layer and SLP lattice that may accommodate additional surface-associated proteins. Four Ca^2+^ ions stabilize loop regions essential for intermolecular interactions and lattice integrity, while their removal reduces overall stability it does not abolish assembly. Comparative analysis with related SLPs, including SbsB from *G. stearothermophilus* and Sap/EA1 from *Bacillus anthracis*, supports a conserved ring-like domain arrangement across Bacillus-type S-layers. Given the involvement of S-layers in host interactions, immune evasion, and pathogenicity, particularly in *Bacillusanthracis* and *Bacillus cereus* group species, the identified intermolecular interfaces represent attractive targets for the development of novel antibacterial strategies.

Crystalline bacterial cell surface layers, termed S-layers, are among the most abundant cellular proteins present in a wide range of bacteria and archaea and are considered additional components of the cell envelope that have emerged during evolution (1, 2). S-layers are often composed of a single secreted protein or glycoprotein species that self-assembles to form a two-dimensional crystalline array on the cell surface (1, 2, 3, 4, 5).

Previous studies involving electron microscopy revealed isoporous lattices with oblique (*p*1, *p*2), square (*p*4), or hexagonal (*p*3, *p*6) symmetry with a center-to-center spacing of the morphological units of approximately 3 to 35 nm (2, 5, 6).

Owing to their intrinsic tendency to crystallize, S-layer proteins (SLPs) reassemble into regular arrays, not only *in vivo* but also *in vitro*, upon removal of the disrupting agent (*e*.*g*., urea, guanidine hydrochloride, pH shift) used in the dissociation procedure. Self-assembly (SA) products generated in suspension may have the form of flat sheets, open-ended cylinders or closed vesicles (5, 7, 8). These general principles of S-layers led to a broad spectrum of applications in the field of nanobiotechnology (5, 7, 8, 9, 10, 11).

Since prokaryotes carrying S-layers are ubiquitous in the biosphere and inhabit the most diverse ecological niches, their surface structures fulfill a broad spectrum of functions. S-layer lattices function as protective coats and are involved in cell adhesion and in the recognition of surfaces in the environment, such as host tissues and abiotic substrates (12, 13, 14). Moreover, S-layers can act as molecular sieves, molecule and ion traps, virulence factors in pathogenic organisms, and as the sole cell wall component in many archaea (2, 5, 15, 16, 17, 18, 19, 20, 21). A general feature of all S-layers is their excellent antifouling property (5).

The cell surface of *Geobacillus stearothermophilus* ATCC 12980 is completely covered by the oblique lattice forming SLP SbsC (22), which consists of 1099 amino acids, including a 30 amino acid long signal peptide (UniProt O68840) (23, 24). These studies revealed that the α-helical N-terminal part comprising amino acids 31 to 258 is exclusively responsible for cell wall binding, whereas the larger, C-terminal part comprises the SA domains responsible for the formation of the crystalline array. In addition, the specific binding mechanism between the positively charged N-terminal region of SbsC and the negatively charged secondary cell wall polymer (SCWP) has been described (25, 26). SCWP-mediated anchoring of S-layer subunits to the cell wall layer guarantees a defined orientation for incorporated S-layer proteins, while allowing enough flexibility for the recrystallization of subunits to continuously assume a low free energy arrangement (2, 3, 5, 7). In different strains of *G. stearothermophilus* the presence of an S-layer is common, although it shows remarkable heterogeneity in molecular weight, geometry and lattice constants (27).

Since SLPs self-assemble into two-dimensional crystals (4, 5), various approaches have been successfully applied in structural studies. The 3D-crystallization of the full-length SLP in the presence of a nanobody as a crystallization chaperone was reported for SbsB (28), SLP from *G.* *stearothermophilus* PV72/p2 and for Sap/EA1, two SLPs from *Bacillus anthracis* (29, 30). The formation of the two-dimensional crystalline assembly was prevented by nanobody-S-layer interaction (28, 30). Recent studies focusing methodologically on cryo electron microscopy also reported full length assembly models for the SLPs of *Clostridioides difficile, Corynebacterium glutamicum, Deinococcus radiodurans, Caulobacter crescentus, Haloferax volcanii, Sulfolobus acidocaldarius, and Nitrosopumilus maritimus* (31, 32, 33, 34, 35, 36, 37). Additionally, the surge of structure prediction reliability led to the development of assembly prediction methods using AlphaFold (AF)-Multimer (38, 39). For example, SymProFold uses different multimer predictions of protein subchains to generate S-layer assembly models without prior knowledge of symmetry or unit cell parameters (38).

For Bacilli S-layers, crystallization of truncated and soluble S-layer constructs has been reported for N-terminal and C-terminal parts of SbsC from *G.* *stearothermophilus* ATCC 12980 (26, 40, 41, 42), as well as the SCWP-binding domain (43) and self-assembly relevant domains of the SLP Sap from *B.* *anthracis* (44). Further analysis of multiple S-layer protein fragments and alternative structural methods enable for the validation of self-assembly models and provide insight into the dynamic behavior of S-layers.

Here we report on high-resolution structures of SbsC fragments containing self-assembly domains. Together with electron crystallography and cryo electron tomography data of SbsC two-dimensional (2D) crystals as well as SymProFold predictions, we propose a model for the self-assembly of the full-length protein, discuss the role of calcium ions and propose self-assembly architecture for related Bacilli S-layer proteins.

## Results

### The structure of the S-layer building block SbsC

A fragment-based crystallographic approach allowed us to determine the complete high-resolution structure of the SbsC S-layer protein (Figs. 1, S1 and Table S1). To obtain the full model, we determined nine X-ray structures of eight SbsC fragments encompassing all domains required for self-assembly, including the linker regions. Only the structure of the N-terminal fragment rSbsC_(31-443)_ was reported previously (26). The first (N-terminal) domain adopts an entirely α-helical fold, whereas the remaining eight domains are rich in β-strands. Topological analysis of individual domains 2 to 9 revealed only limited similarity to Ig-like domains from a variety of proteins from different origin, including the X-ray structures of another S-layer protein from *G.* *stearothermophilus* SbsB (28) and Sap from *B.* *anthracis* (30).

**Figure 1:**
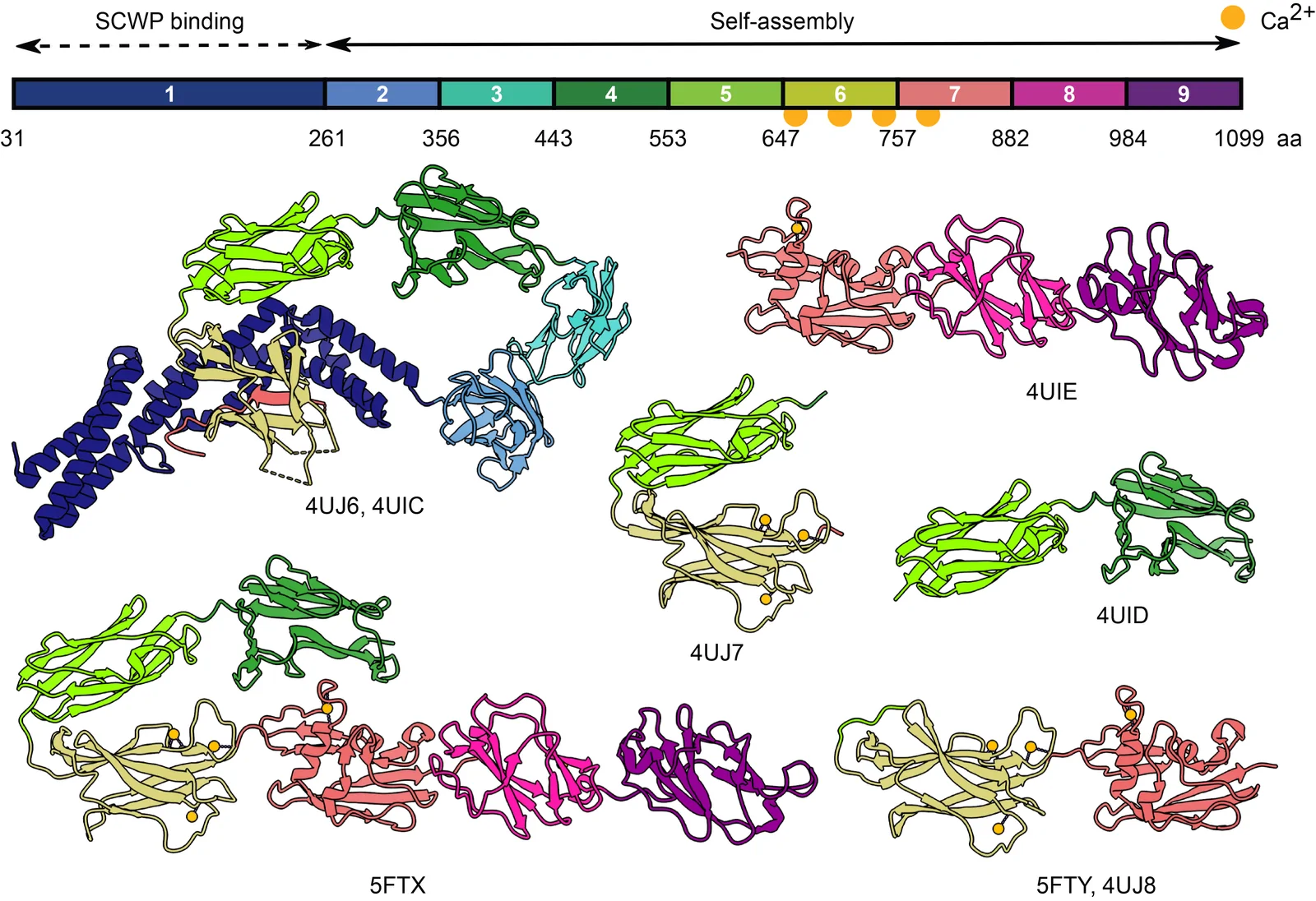
**X-ray structures of *G.ste******arothermophilus*****SbsC fragments.** Graphical representation of full-length rSbsC with indicated domain borders (domain 1–*midnight blue*, domain 2–*steelblue*, domain 3 –*light seagreen*, domain 4–*forestgreen*, domain 5–chartreuse, domain 6 – *dark khaki*, domain 7–*indian red*, domain 8–*deep pink*, and domain 9–*purple*). The secondary cell wall polymer -binding domain providing cell wall attachment and the self-assembly regions are indicated by a *dashed* and *solid arrow* respectively. Ca^2+^ sites are indicated with *orange spheres*. Crystal structures are depicted in cartoon representation: 4UJ6: rSbsC_(31-790)_; 4UIC: rSbsC_(31-844)_; 4UIE: rSbsC_(754-1099)_; 4UJ7: rSbsC_(541-759)_; 4UID: rSbsC_(443-650)_, 5FTX: rSbsC_(447-1099)_, 5FTY, 4UJ8: rSbsC_(642-882)_.

Four sevenfold coordinated calcium ions are situated in the loop regions of domains 6 and 7 (Figs. 1, S1 and S2). Two calcium ions are accommodated in the first loop region of domain 6 at a distance of 9.4 Å. The third calcium site is positioned in another loop of domain 6. An additional calcium ion is situated in the loop region of domain 7. Thermostability of SbsC fragments containing domains 6 and 7 is significantly increased when Ca^2+^ is present in solution (Fig. S3 and Table S2).

### Self-assembly

Previous study with truncated fragments of SbsC showed that the removal of the first domain, responsible for the cell wall binding, and the last two domains (domains 8 and 9) do not interfere with the self-assembly process (24). To explore the conformation of SbsC in its self-assembled form, 2D crystals of full-length SbsC and N- and C-truncated fragments (rSbsC_(258-1099)_ and rSbsC_(31-920)_) were produced and studied by electron crystallography and cryo electron tomography (cET) (Fig. S4). By electron crystallography, mostly double-layered flattened cylinders and some sheet-like crystals were observed and no additional Ca^2+^ was needed to obtain the 2D crystals. 2D crystals of both truncated fragments showed, on average, slightly lower diffraction quality and reproducibility than the full-length SbsC. Nonetheless, the unit cell dimensions of all three samples were the same: a = 108 Å, b = 72 Å and γ = 82 ° with *p*1 symmetry.

Subsequently, cET of flattened cylinders provided the three-dimensional organization of SbsC within the tubular array. The lattice parameters were virtually the same as determined by 2D crystallography and individual layers exhibited *p*1 symmetry (Fig. 2). Each layer consisted of a core region (Fig. 2*B*, marked c in insert), and two additional regions (Fig. 2*B* marked i and o in insert) pointing inwards and outwards.

**Figure 2:**
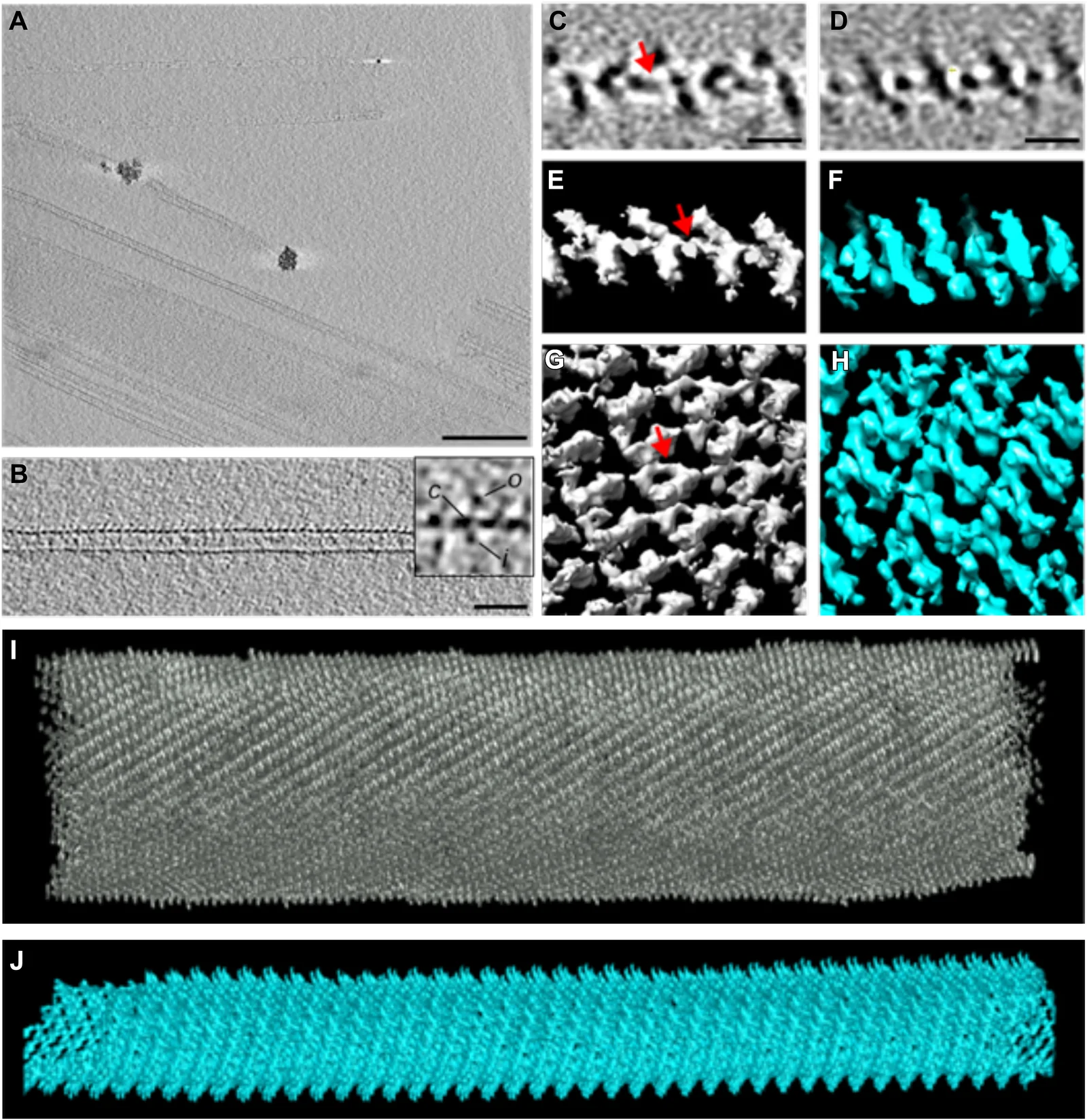
**Cryotomography of the reassembled *G.ste******arothermophilus*****S-layer.***A*, cryotomography slice through *G.stearothermophilus* S-layer tubes in the presence of CaCl_2_. The scale bar represents 200 nm. *B*, cross section through double-layered S-layer tube from *A*. The insert shows the densities belonging to the core-region (*c*) and the inwards (*i*) and outwards (*o*) regions, on the inside and outside of the tube, respectively. Scale bar 50 nm. *C* and *H*, subtomogram averages of reassembled SbsC in the presence (*C*, *E* and *G*) and absence (*D*, *F* and *H*) of CaCl_2_. *C* and *D*, slices through the subtomogram averages. *E* and *F* and *G* and *H* are surface representations of side views and top views respectively. *Red arrows* in *C*, *E* and *G* indicate the core region harboring the Ca-ions. The scale bars represents in *C* and *D*, 10 nm. 3D models of the reassembled *G.stearothermophilus* S-layers in the presence (*I*) and absence (*J*) of CaCl_2_. The models were obtained by plotting the averaged volumes back into the original cryotomograms.

Addition of CaCl_2_ was not essential for reassembly of full-length SbsC into crystals. However, cET revealed structural differences between lattices reassembled in the presence and absence of CaCl_2_ (Fig. 2, *C–H*). Two sub-tomogram averages were obtained from tomograms of S-layers reassembled with CaCl_2_ and without CaCl_2_. The average obtained from samples with CaCl_2_ shows the additional density in the core-region harboring the calcium ions (Fig. 2, *C*, *E* and *G*, red arrows). In the S-layer, this additional density is not visible in the average obtained without CaCl_2_ (Fig. 2*D*, *F* and *H*). This is in agreement with our observation that good diffraction patterns and better thermostability for fragments containing domains 6 and 7 were obtained in the presence of CaCl_2_, indicating that Ca^2+^ has an important role in correct positioning of the loop regions and domain stabilization.

### Ring-like formation of SbsC monomer

Fitting of the atomic structures into the tomographic data proved to be challenging since apparent flexibility in the S-layer assembly reduced the overall resolution of the subtomogram averages. Nevertheless, the repetitive ring-like structures are recognizable in the subtomogram averages of SbsC in the presence of Ca^2+^ (Fig. 2*G*).

Superposition of determined X-ray structures containing various SbsC fragments (Fig. 1) and the subtomogram average from S-layers with added Ca^2+^ enabled us to substantiate AlphaFold 3 based SymProFold predictions for full-length SbsC present in the self-assembled state (Figs. 3*A* and S5). The protein exhibits an ellipsoid shape, with its core region (domains 2–7) showing a similar size to that observed in the subtomogram average of the Ca^2+^-containing S-layer. In the assembled S-layer, intramolecular contacts between domain 2 and 7 are relevant for ring closure. The ring-domains thus form the main plane of the S-layer, with the SCWP binding domain 1 projecting below this plane. In contrast, the flexible domains 8 and 9 protrude above the plane into the extracellular space. The presented crystal structures did not indicate any relevance of domains 8 and 9 for SA, supporting their designation as an auxiliary region, as previously reported (23).

**Figure 3:**
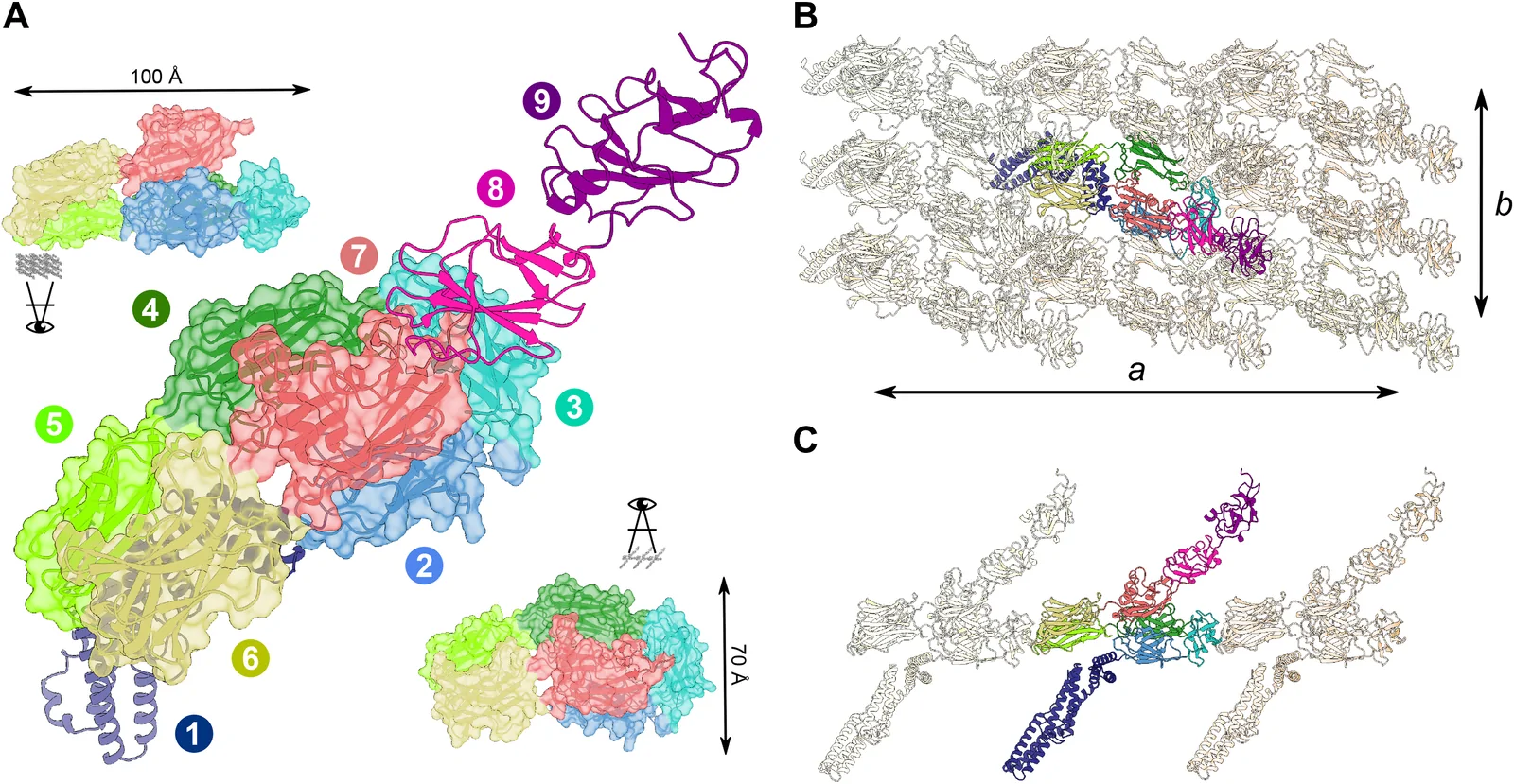
**Full length model and proposed self-assembly of SbsC.***A*, monomer model from AlphaFold 3 predictions compliant to crystal structures. SA relevant core domains are shown in surface representation. The secondary cell wall polymer binding domain 1 and the auxiliary domains 8 and 9 are shown in cartoon representation only. *Side* and *top* view of the SA core domains in the corners (*upper left corner*: *side view*, *lower right corner*: *top view*). *B* and *C*, *top* and *side view* of SymProFold prediction for SbsC for a 3 × 3 tile with one monomer highlighted in colored domains. The *a* and *b* directions of the self-assembled lattice are indicated with *arrows* and correspond to the unit cell parameters of the 2D-lattice (a = 108 Å, b = 72 Å and γ = 82 ° with *p*1 symmetry). SA, self-assembly.

Superposition of available structures indicates that some linker regions show more rigidity than others. Linker regions connecting domains 4 to 5 and domains 5 to 6 are congruent in different crystal structures, resulting in defined orientations that appear mandatory for maintaining the ellipsoidal shape. Domain 7 is positioned in the vicinity of domain 2, thus closing the ring. In this arrangement, additional intra-molecular interactions between domains 4 and 7, as well as domains 5 and 6 are formed (Fig. S6). In contrast, linkers between domains 1 to 2, 2 to 3 and 6 to 7 exhibit more diverse conformations (Fig. S7). This allows for more flexibility of adjacent domains involved in efficient binding to the SCWP (domain 1) and for interactions of the auxiliary region with the environment.

### SbsC self-assembly model and intermolecular interactions

On the basis of the available crystal structures of SbsC fragments and considering the essential role of the ring domains 2 to 7 in self-assembly, we analyzed the interactions within the crystal packing. We observe the repetitive in-plane translation in two directions, corresponding to two-dimensional crystal lattice directions *a* and *b* (Fig. 4, *A*–*C*). The domains of neighboring SbsC monomers involved in these intermolecular contacts are domains 3 to 6 in *a* direction (present in PDB 4UIC and 4UJ6) and 4 to 7 in *b* direction (present in PDB 5FTX). Both contact regions are defined by hydrophobic patches (Fig. 4, *E* and *G*) and in the case of 4 to 7 also intermolecular hydrogen bonds. The interaction 3 to 6 involves residues in the regions 402-404/422-438 with 652-653/743-748 with hydrogen bonds from S403 to T745 and V746 and from N436 to T747 (Fig. 4*D*). Domains 4 to 7 include residues in the regions 824-827/858-873 and 513-528, respectively (Fig. 4*F*).

**Figure 4:**
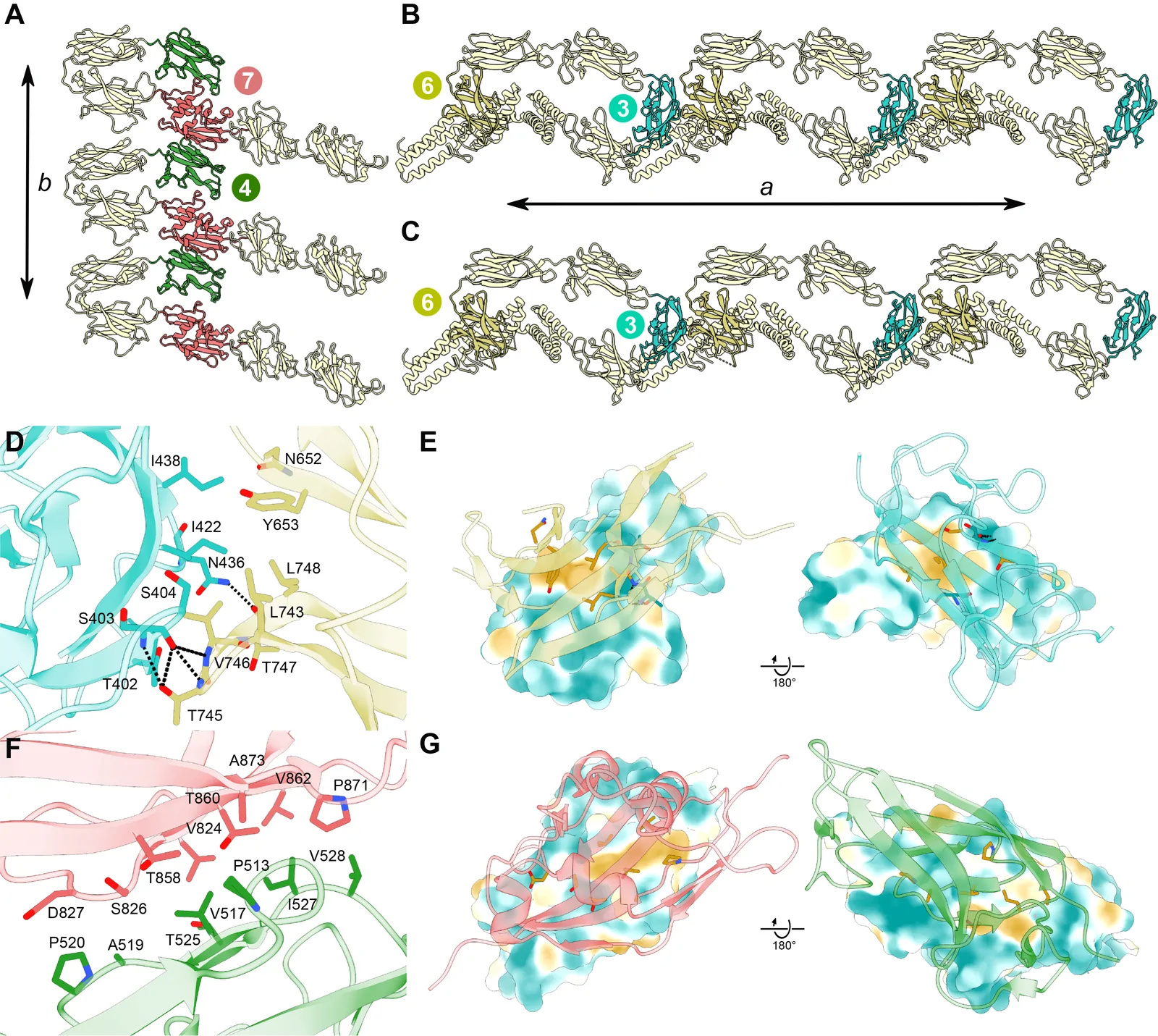
**Intermolecular contacts as observed in the experimental crystal structures.***A*, translation in *b* direction through intermolecular contacts between domains 4 (*forest green*) of one SbsC monomer and domain 7 (*indian red*) of the neighboring monomer is observed in rSbsC_(447-1099)_ (PDB 5FTX). *B* and *C*, crystal packing as observed in (*B*) rSbsC_(31-844)_ (PDB 4UIC) and (*C*) rSbsC_(31-790)_ (PDB 4UJ6). Translation in *a* direction through intermolecular contacts between domain 3 (*light seagreen*) of one SbsC monomer and domain 6 (*dark khaki*) of the neighboring SbsC monomer. The *a* and *b* directions of the self-assembled lattice are indicated with *arrows* and correspond to the unit cell parameters of the 2D-lattice (a = 108 Å, b = 72 Å and γ = 82 ° with *p*1 symmetry). *D*, zoom in of the intermolecular interaction surface between two neighboring monomers, showing interacting residues (in sticks) in *a* direction including domain 3 (*light seagreen*) from one SbsC monomer and domain 6 (*dark khaki*) from the second SbsC monomer, as present in the crystal structure rSbsC_(31-844)_ (pdb 4UIC). *E*, interaction between domain 3 (*light seagreen*) and domain 6 (*dark khaki*) shown with each domain once in cartoon representation and the other in a surface representation colored by hydrophobicity (ChimeraX mlp, *dark cyan*-hydrophilic, *dark goldenrod* - hydrophobic), highlighting hydrophobic properties of the interaction interface. *F* equivalent to *D* for intermolecular interaction surface between two neighboring SbsC monomers in *b* direction including domains 4 (*forest green*) from the first SbsC monomer and domain 7 (*indian red*) from the second SbsC monomer as present in the crystal structure rSbsC_(447-1099)_ (pdb 5FTX). *G* Equivalent to *E*, corresponding to individual domains 4 (*forest green*) and 7 (*indian red*).

Performing SymProFold predictions using the full-length SbsC sequence as input, a plausible SA mechanism can be proposed maintaining *p*1 symmetry as well as the intermolecular contacts between domains 3 to 6 and 4 to 7 (SymProFold: a = 99 Å, b = 50 Å, γ = 89°, EM: a = 108 Å, b = 72 Å, γ = 82°). Translation along the *a* and *b* directions is equivalent to the ones observed in the crystal contacts and variation of relative domain arrangement is well within plausible linker flexibility (Fig. 3, *B* and *C*). The model shows domain packing that reduces the pore diameter relative to the experimental structure which leads to the discrepancy of the cell dimensions obtained by SymProFold and EM. This model reflects the obtained 2D projection from 2D crystals and subtomogram averages in the core region. The flexibility of domain 1 on one side and 8 to 9 on the other is also observed in the subtomogram averages, where no clear and repetitive density within these regions is present (Fig. 2). Mapping of the electrostatic potential onto the assembled SbsC layer reveals more negatively charged patches on the surface exposed to the environment (Fig. S8). Positive charges are observed in the SCWP-binding region of domain 1 (Fig. S8).

### Structural comparison with S-layer proteins SbsB and Sap

Experimental structures of three related S-layer proteins, SbsB from *G.* *stearothermophilus* (PDB 4AQ1), as well as Sap and EA1 from *B.* *anthracis* are available and show structural similarities (PDB 6QX4, 6HHU, 8OPR, 9G93) (28, 29, 44, 45). In *B.* *anthracis*, two different SLPs, Sap and EA1, are expressed in a cell cycle-dependent manner (46). All these S-layer proteins share a common architecture, consisting of an N-terminal cell wall binding domain followed by several Ig-like domains connected by short linkers. Pairwise structural alignment shows that the domain order in all four proteins is analogous (Fig. S9). The SCWP binding domain of SbsC shows a unique fold mainly containing α-helices (26), in comparison to the SLH family domain (47) reported for SbsB, Sap and EA1. The auxiliary region of domain 8 and 9 is unique to SbsC and domain 7 varies most in size and structure (Fig. 5). The reported and herein proposed structures of the SA core domain show that the monomer arranges in an elongated ring-like structure with a hinge between domain 5 and 6 and rather linear arrangement for domains 4 to 5 and 6 to 7. All monomers form a rather flat core parallel to the membrane plane (Fig. 5*B*, side view). Striking plasticity is visible in the arrangement of the first three domains, giving unique ring-closure mechanism for each protein (Fig. 5*B*). Regarding the assembled layer one has to keep in mind that Sap shows *p*2 symmetry, while the other proteins EA1, SbsB and SbsC crystallize in *p*1 symmetry. Therefore, the translation along *a* is uniquely characterized in Sap by two different 2-fold symmetrical interfaces, where the remaining assemblies are defined by only one distinct interaction surface each. Translation along *b* is conserved in all compared S-layers and involves intermolecular interactions between domains 4 and 7 (Fig. 5*C*), which we analyze in more detail below.

**Figure 5:**
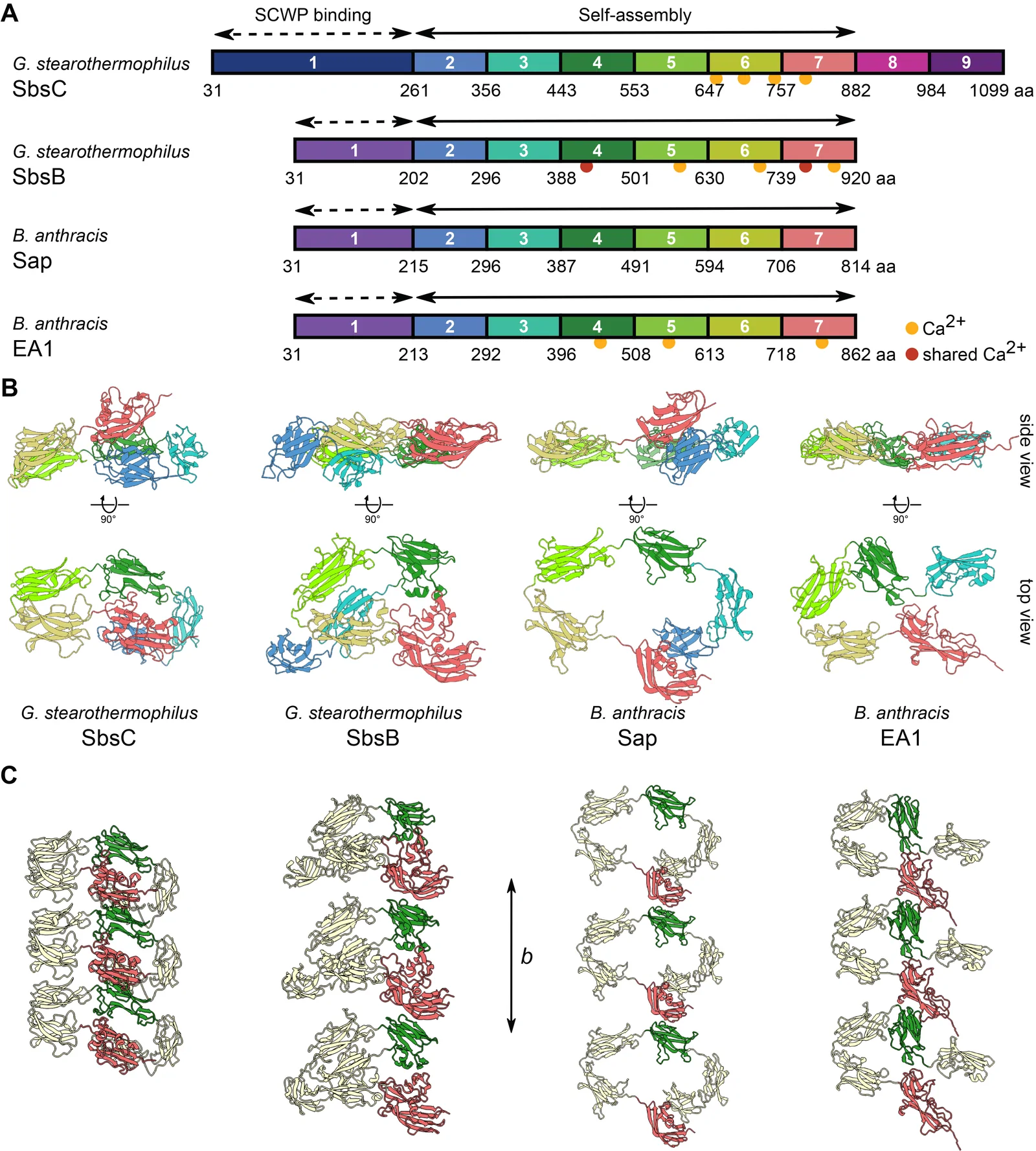
**Domain organization and SA forming unit of related SLPs.***A*, domain organization scheme for SbsC and SbsB from *G.stearothermophilus* and SAP and EA1 of *B.anthracis* with major differences in the cell wall attachment domain and missing auxiliary domains in all but SbsC. Ca^2+^ binding sites are shown as *orange/red spheres*. *B*, *top* and *side* view of the SA core domains for all bacilli SLPs with described structure. SbsC: this work, predicted full length model validated with different domain structures; SbsB: pdb 4AQ1, cocrystallized SLP with nanobodies; Sap: pdb 9G93, reconstructed from cryoET; EA1: reported model by Sogues *et al.* (29, 45), reconstructed from cryoEM. *C*, three repeating SA-units for the proteins SbsC, SbsB, Sap and EA1 along direction *b* with highlighted domains 4 (*forest green*) and domain 7 (*indian red*), as observed in available crystal structures (SbsC: pdb 5FTX, SbsB: pdb 4AQ1, Sap: pdb 9G93) or published model by Sogues *et al.* for EA1 (29, 45). SA, self-assembly.

### Interfaces between domain 4 and 7 cluster in two distinct types

To determine whether the conserved domain 4 to 7 interface observed in SbsC, SbsB, Sap, and EA1 extends to other *Bacillus* S-layer proteins lacking experimental structures, we analyzed sequences and predicted structures of Sap/EA1 homologs. Sequence homologs can be found in related species like *Bacillus cereus*, *Bacillus thuringiensis* and others. Sequence alignment only shows high identity for EA1 homologs and Sap homologs but not between EA1 and Sap-type proteins or to either SbsB or SbsC (Fig. 6*A*, yellow and orange boxes). This holds true for the full-length sequences, as well as for the single domains 4 and 7 (Fig. 6*A*). Nevertheless, a structural homology and a conserved domain organization are present at least for representatives of different types (Sap and EA1), and different source organisms (*B.* *anthracis* and *G.* *stearothermophilus*) (Fig. 5*B*). To extend this analysis to the structural level, AlphaFold 3 predictions were performed using full-length sequence together with an additional sequence corresponding to either domain 4 or 7 of Sap and EA1 from *B. cereus* and *B. thuringiensis* (Bcer_EA1, Bcer_Sap and Bthu_EA1, Bthu_Sap). Alignment of the highest-scoring predictions to the crystal structure of Bant_Sap domain 4 revealed two distinct types of domain 4 to 7 interaction surfaces (Fig. 6*B*): a Sap-type interface, shared by Sap, SbsC and SbsB, and an EA1-type interface, characteristic of EA1 homologs. The SbsB and SbsC proteins from *G.* *stearothermophilus* display a relatively small domain 4 to 7 interaction interfaces that combines features of both Sap- and EA1-type interactions, with a domain orientation more closely resembling the Sap cluster. In all cases, the interfaces are dominated by nonspecific hydrophobic contacts with only a few hydrogen bonds.

**Figure 6:**
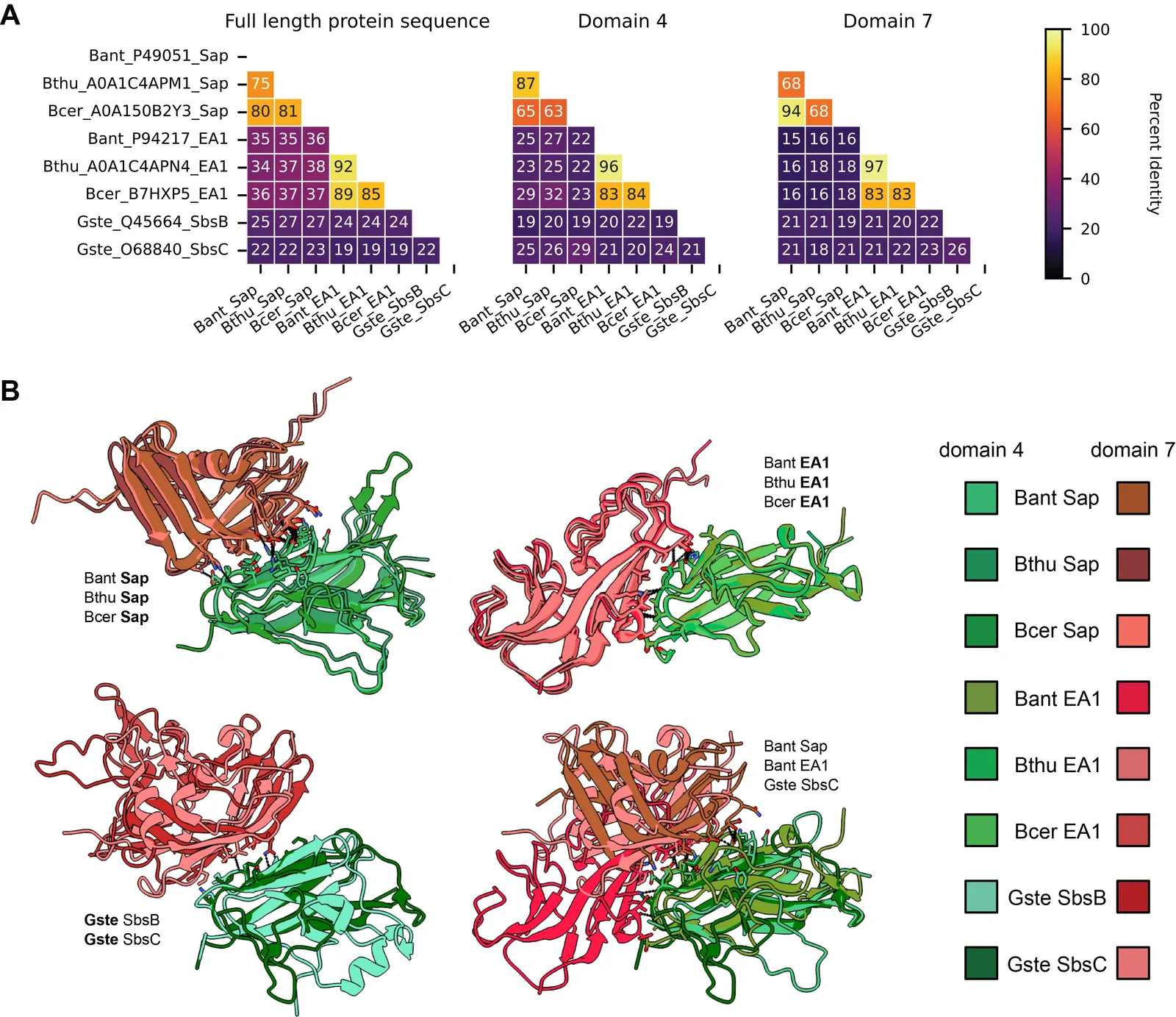
**Structural and sequence homology of SA contact between domain 4 and 7.***A*, percent identity matrices for sequence alignments of SLPs from related Bacilli *B.anthracis*, *B.thuringiensis*, *B.cereus* and *G.stearothermophilus* for either the full-length sequence (UniProt identifier is indicated) as well as separate alignments for domains 4 and 7 (visualization with matplotlib and seaborn). *B*, structural alignments of the interface between domain 4 and 7 shown for Sap homologs, EA1 homologs, SbsB and SbsC, as well as both *B.anthracis* and SbsC proteins. Alignment is performed with ChimeraX matchmaker for domain 4 to domain 4 of *B.anthracis* Sap; contacting residues are shown in *sticks* representation with pseudobonds for H-bonds in *black*; *B.anthracis* Sap: 9G93, *B.anthracis* EA1: published model by Remaut *et al.* (29, 45), *G.stearothermophilus* SbsB: 4AQ1, *G.stearothermophilus* SbsC: 5FTX, remaining: AlphaFold 3 predictions of the full length proteins and an additional domain 4 and domain 7. Bant, Bacillus anthracis; Bcer, *B. cereus*; Bthu, *B. thuringiensis*; Gste, G. stearothermophilus; SA, self-assembly.

## Discussion

### Curvature, sub-S-layer space, and structural adaptability

In nature, the bacterial cell wall acts as a scaffold for the S-layer proteins, providing a surface for the proteins to self-assemble into a regular lattice structure. As the bacterial cell grows, the S-layer proteins adjust their conformation to maintain their attachment and position on the cell surface. This proposed general layout is enabled by the ring domains, which are stabilized by a mix of rigid and flexible linker regions.

Studies in Geobacillus/*Bacillus* demonstrated that new S-layer subunits are inserted locally at the cell surface and can migrate laterally to re-establish long-range crystalline order, with lattice orientation tracking the cylindrical axis of the cell. These *in vivo* observations underscore that inter-subunit cohesion within the lattice exceeds the affinity to the SCWP, enabling continuous re-crystallization into a low free-energy state during cell elongation and division (1, 5, 48). Our ring-like model with defined rigid and flexible linkers provides a structural rationale for such behavior. While robust lateral interfaces (notably the domain 4–7 interface) maintain lattice order, flexible hinge/linker regions between domains 1 to 2, 2 to 3 and 6 to 7 (Fig. S7) allow curvature matching and lattice reorganization during growth. This suggests a structural mechanism by which individual lattice tiles can undergo small bending or tilting movements to follow the curvature of the cell surface while preserving local packing. Flexibility in the domain 1 to 2 linker further facilitates adaptive attachment to the SCWP and peptidoglycan layer without perturbing the two-dimensional symmetry of the S-layer. This ability of S-layers to conform to the curvature of the cell surface is important for their functions such as stability, protection and cell adhesion, as it allows the bacteria to interact with a variety of surfaces and the environment. In addition, the size of the cell wall binding domains, especially in the example of SbsC, indicates that, upon self-assembly on the bacterial surface, the S-layer lattice creates a space between the peptidoglycan layer and the assembled core region. We refer to this enclosed volume as the sub-S-layer space. This space can potentially accommodate also larger molecules like other surface-associated proteins.

Beyond curvature matching and sub-S-layer space, our SbsC model also underscores an architectural distinction from many previously characterized S-layers: in most systems, pores emerge primarily at interfaces between neighboring S-layer monomers, whereas in SbsC the pores are largely defined within individual subunits by the ring-like arrangement of domains 2 to 7. In such an architecture, pore geometry is encoded at the level of a single polypeptide rather than being determined mainly by the relative packing of adjacent subunits. This organization could enable greater evolutionary flexibility in tuning pore diameter and shape through intra-molecular changes (*e*.*g*., in ring closure and linker geometry), while preserving the overall lattice topology. In turn, this may facilitate independent optimization of pore size, charge distribution and curvature adaptability to balance permeability, protection and antifouling properties. This model is consistent with molecular sieving studies in *G.* *stearothermophilus* S-layers, which permit free diffusion of proteins in the 20 to 45 kDa range (estimated pore diameter ∼4.5 nm), including exoenzymes and lytic enzymes, thereby supporting secretion and turnover at the cell surface (49). The large pores and the low propensity for nonspecific adsorption in Bacillaceae S-layers foster efficient nutrient/metabolite exchange and minimize clogging, features essential for sustained growth and for biotechnological exploitation of S-layers as ultrafiltration membranes with uniform nanopores (49, 50). Furthermore, net charge differences between the S-layer and the underlying sacculus, together with the low propensity for nonspecific macromolecular adsorption in pore areas, modulate surface recognition and nutrient exchange (49, 51).

### Dynamic self-assembly, lattice plasticity, and antifouling

S-layer lattices display intrinsic dynamics at the level of morphological units, as seen in freeze-etch preparations where many units appear slightly twisted relative to ideal lattice positions despite rapid vitrification (1, 5, 52). This suggests oscillatory motions within the array that are frozen at different stages during sample preparation. Such dynamic plasticity is consistent with our observation of flexible linkers enabling curvature accommodation and may contribute to the antifouling properties of S-layers by minimizing nonspecific macromolecular adsorption onto the surface (5). The interplay between rigid ring-closure elements (*e.g.*, SbsC domains 5–6) and locally flexible linkers likely underpins both long-range order and local motility required for surface adaptability and self-cleaning behavior.

### Calcium/ion effects and lattice stabilization

Our cryo-ET and thermostability assays indicate Ca^2+^-stabilized loops in SbsC domains 6 to 7 that are visible in subtomogram averages and enhance diffraction quality of constructs containing these domains. While CaCl_2_ is not strictly required for reassembly, it improves stability. Notably, ion substitution (*e*.*g*., Fe^2+^ for Ca^2+^) has been shown to induce assembly in related S-layer systems (53), suggesting potential ion-specific modulation of loop conformations and assembly kinetics.

Across Bacillaceae S-layers, Ca^2+^ plays protein-specific roles. SbsB shows Ca^2+^-triggered assembly (28). For SbsC, Ca^2+^ stabilizes loop regions in domains 6 to 7 and enhances thermostability and diffraction but is not essential for assembly. In *B.* *anthracis*, EA1 harbors divalent-ion binding sites and its lattice formation is facilitated and stabilized by Ca^2+^ under commonly used *in vitro* reassembly conditions, though strict Ca^2+^ dependence similar to SbsB has not been universally demonstrated (29, 44, 45). Sap assembles without obligatory Ca^2+^ (30, 44). Where present, Ca^2+^ sites cluster in exposed loop regions of the assembly core and act as a tunable stabilizer or trigger across Bacillus-type S-layers.

### General S-layer architecture across bacillaceae

Based on our proposed model of SbsC and similarities to Sap and EA1, we suggest a general layout for S-layer protein assemblies in related species in the *B.* *cereus* group, potentially transferable to other Bacillaceae. AlphaFold 3 predictions of Sap/EA1 homologs in *B.* *cereus* and *B*. *thuringiensis* highlighted recurrent contact motifs, notably the domain 4 to 7 interface. These cross-species predictions support a generalized assembly scheme and provide an *in silico* map of symmetry-dependent interfaces that can be experimentally validated.

The proposed self-assembly consists of a central core region exhibiting 6 Ig-like domains (domains 2–7) arranged in a closed ring-like formation, and an anchoring domain 1 at the N-terminus, located beneath the central domain. The translation in *b* direction occurs *via* the interactions between domains 4 and 7 of the neighboring monomers. The disruption of the interface between domains 4 and 7 can serve as a general target for the interference with the S-layer assembly. This is particularly relevant for pathogenic bacteria like *B*. *antracis, B.* *cereus* *and B. thuringiensis*. In the absence of comprehensive experimental data on the symmetry of all S-layers from this group (*p*1, *p*2, or even higher symmetry), it is challenging to establish any general rules for the translation in *a* direction or the occurrence of dimerization.

## Pathogenic implications and targeting assembly interfaces

In the context of interactions with human hosts, S-layers play a critical role in bacterial pathogenesis and adaptation. In members of the *B.* *cereus* group, they facilitate adhesion to extracellular matrix components and intestinal epithelial cells (54, 55, 56), and can trigger inflammatory responses such as intraocular inflammation (57). They also interact directly with immune cells, enabling bacteria to evade both innate and adaptive immune mechanisms (58) and have been suggested to play a role in biofilm formation (59).

Recently, Fioravanti *et al.* demonstrated that disrupting the protein–protein interactions (PPIs) required for S-layer assembly in *B.* *anthracis* using nanobodies prevented lethality in a mouse anthrax model (30), highlighting the S-layer as a promising antibacterial target. Although PPIs are classically challenging, recent advances have yielded numerous small-molecule PPI inhibitors, including at dynamic or transient pockets revealed by conformational sampling and fragment-based discovery (60, 61, 62). Mapping interface hotspots at the domain 4 to 7 and domain 3 to 6 contacts provides concrete targets for cross-species interference within the *B.* *cereus* group, offering a route to broad-spectrum antivirulence strategies that weaken surface armor without necessarily imposing strong resistance selection (28, 29, 30, 44, 45, 60, 61, 62). Nanobody- or peptide-based inhibitors can be designed to target interfaces that are specific to each lattice symmetry, thereby maximizing selectivity. Such strategies show potential for the development of therapeutics against pathogenic *Bacillus* species, including *B.* *cereus* which, in addition to causing foodborne illness (emetic and diarrheal syndromes), can lead to severe systemic infections such as bacteraemia, endophthalmitis, and pneumonia, particularly in vulnerable individuals (63).

In conclusion, the structural characterization of SbsC reinforces a modular architecture conserved across *Bacillus* S-layers: an anchoring domain connected to an assembly module consisting of six Ig-like domains forming tile-based lattices that adapt to cell curvature and create a sub-S-layer space. Comparative analysis with Sap/EA1 of *B.* *anthracis* and *B.* *cereus* and with SbsB highlights conserved assembly mechanisms (inter-domain contacts) and suggests a generalizable assembly scheme across *Bacillus* species.

Disrupting critical inter-domain interactions, particularly in conserved contact regions between domain 4 and 7 of ring-like Bacilli SLPs, offers a rational strategy for antivirulence therapies, potentially applicable across pathogenic Bacilli. From an antimicrobial perspective, targeting subunit supply, secretion, or SCWP binding in tandem with PPI disruption may lower the threshold for S-layer collapse in pathogens.

Future high resolution structural studies (*e.g.* cryo-ET reconstructions supporting SymProFold predictions) combined with dynamic simulations could reveal transient pocket formation and further enable rational inhibitor design.

## Experimental procedures

All chemicals were purchased from Sigma-Aldrich or Carl Roth GmbH, if not stated otherwise.

### Cloning, expression and purification of SbsC fragments

Protein expression and purification of all fragments was conducted according to published protocols (24, 40, 41, 42). In short, the recombinant fragments were expressed in *Escherichia coli*, the soluble fraction after lysis was subjected to a fractionated ammonium sulfateprecipitation. The pellet was dissolved in 2 M guanidine hydrochloride, 150 mM NaCl, 50 mM Tris pH 7.2 and dialyzed against distilled water. After centrifugation, the supernatant was applied onto a Superdex 200 column. SbsC fragment-containing fractions were pooled and concentrated for crystallization.

### 3D-crystallization of soluble SbsC fragments

In order to prevent the 2D-self-assembly we produced soluble truncated fragments of the SbsC protein. The crucial step in obtaining suitable crystals for structure determination proved to be the correct definition of domain borders. For some fragments, this was achieved by *in situ* proteolysis (40, 41). The Table S1 gives an overview about the 3D-crystallization and structure determination of all SbsC fragments. Crystallization of fragments rSbsC_(443-650)_, rSbsC_(541-759)_, rSbsC_(31-790)_ and rSbsC_(755-1099)_ is described elsewhere (40, 41). Initial crystal screening for all other constructs was done with an Oryx8 robot using Douglas vapor-batch plates (Douglas Instruments). Drops of 1 μl were dispensed in 1:1 ratio of protein and commercial screening solution. Commercially available screens Index, Crystal Screen 1 and 2 (Hampton Research), Morpheus and JCSG+ (Molecular Dimensions) were used for initial setups. All plates were incubated at 293 K.

### Crystallization of rSbsC_(642-882)_

Crystals of rSbsC_(642-882)_ appeared after 4 to 5 weeks in several drops of both JCSG+ and Index screen. It crystallized in two different space groups, *P*1 and *P*2_1_, diffracting to 1.7 and 2.5 Å, respectively. The crystallization condition of the best diffracting crystal of triclinic space group was from Index screen condition 70 (0.2 M NaCl, 0.1 M Bis-Tris pH 5.5 and 25% PEG 3350) with protein stock concentration of 9 mg/ml in 50 mM Tris/HCl pH 7.2 and 2 mM CaCl2 buffer. The crystallization condition of the best diffracting monoclinic crystal was JCSG+ screen 2 to 46 (0.2 M ammonium acetate, 0.1 M Bis-Tris pH 5.5 and 25% (w/v) PEG 3350) with protein stock concentration of 5 mg/ml in 50 mM Tris/HCl pH 7.2 and 2 mM CaCl_2_. Data collection was performed at 100 K without additional cryoprotectant.

### Crystallization of rSbsC_(447-1099)_

Crystallization of rSbsC_(447-1099)_ in the presence of 2 mM Ca^2+^ yielded several crystals of different morphology whereas only plate-like crystals showed diffraction. The construct rSbsC_(447-1099)_ was crystallized in the optimized Index screen condition 93 (41). The best diffracting crystal appeared after 5 days in a condition containing 0.02 M zinc acetate dihydrate and 8% (w/v) PEG 3350 (protein concentration of 8 mg/ml and 2 mM CaCl_2_). Stepwise glycerol dehydration of the crystals improved the diffraction. Glycerol was added to the drop (end concentration 20%) and left to equilibrate 1.5 h. After adding glycerol, a big crystal was broken in smaller pieces, which were frozen and tested after 15 to 90 min equilibration time. Best diffraction was gained after equilibrating with glycerol for 60 min showing anisotropic diffraction with high-resolution limits of 3.5 and 5 Å, respectively.

### X-ray data collection and structure determination-rSbsC_(31-790)_, rSbsC_(443-650)_, rSbsC_(541-759)_, rSbsC_(642-882)_ and rSbsC_(447-1099)_

Crystallization and initial data processing of the constructs rSbsC_(31-790)_, rSbsC_(443-650)_ and rSbsC_(541-759)_ are reported elsewhere (40). For rSbsC_(642-882)_ and rSbsC_(447-1099)_ data were collected at the ESRF (Grenoble) and Elettra (Trieste). The data were processed and scaled using the XDS program package (64). Data collection and processing statistics for rSbsC_(642-882)_ and rSbsC_(447-1099)_ data are in Table S5.

Structure determination of these constructs was performed by molecular replacement (MR) using Poly-Ala models of rSbsC_(31-844)_ individual domains (4, 5, 6) as templates (26) in program Phaser (65, 66). Later, structures of rSbsC_(642-882)_ and rSbsC_(447-1099)_ were solved by MR with Phaser using individual domains of high-resolution structures of rSbsC_(443-650)_, rSbsC_(541-759)_ and rSbsC_(755-1099)_ as initial models (PDB: 4UID, 4UJ7, 4UIE). Linker regions were manually rebuilt in Coot (67). Refinement of the structures was performed with REFMAC5 (68) or Phenix (69) followed by manual inspection and model rebuilding in Coot (67). Since rSbsC_(447-1099)_ contains lower resolution data, Phenix-ready set was used to generate metal coordination restraints. Structure validations were performed using MolProbity (70). Complete data collection and refinement statistics are shown in Table S6.

### Crystallization and structure solution of rSbsC_(755-1099)_

For rSbsC_(755-1099)_ native and osmium-derivative data from *P*4_1_2_1_2 crystals obtained in Index condition 27 (2.4 M and sodium malonate pH 7.0) were available (41). Additional native data were collected (Elettra, Trieste) on a crystal belonging to *P*2_1_2_1_2 space group and growing in condition 93 from Index screen (0.05 M zinc acetate, 20% PEG 3350). Heavy atom soaking was proceeded with crystals obtained in Index condition 27, as diffraction was reproducible. An additional derivative data set was collected (EMBL/DESY) on the crystal soaked in 0.5 mM potassium tetrachloroaureate for 5 h. Within this same condition, one crystal with different crystal form was observed. It diffracted to 3.1 Å and belonged to the space group *P*3_1_21. All new obtained data sets were processed using Denzo and Scalepack (71). Data collection and processing statistics for new datasets are given in Table S4.

The initial phasing and phase extension (76% solvent content) was performed using all available data sets from *P*4_1_2_1_2 crystals using MIRAS method by Solve/Resolve (72, 73) within Phenix (69). The obtained electron density map showed clear protein/solvent boundaries and three domains could be identified. A partial poly-Ala model was manually built using Coot (67). The obtained model was divided in three domains that were used as initial templates for MR of the other two crystal forms (*P*3_1_21 and *P*2_1_2_1_2) using Phaser (66). Multi-crystal averaging was performed by DMMULTI within the CCP4 (74) using data of all available forms (*P*3_1_21, *P*4_1_2_1_2 and *P*2_1_2_1_2). Finally, the best electron density map was obtained from a crystal belonging to the space group *P*3_1_21. Additional residues were manually fit within Coot and the obtained model was subjected to 5 cycles of rebuilding and refinement using Buccaneer (75) and REFMAC5 (68). This procedure was continued until all residues were placed in the electron density map.

A search for structural similarity of individual SbsC domains was carried out using PDBeFOLD (76). Superposition of structurally similar domains was performed using SSM Superposition (76) as implemented in the program Coot (67), UCSF ChimeraX MatchMaker (77) and *super* integrated in PyMOL.

### Cryo electron-tomography

Purified samples of rSbsC_(31-1099)_ (1 mg/ml) were mixed 1:1 with 6 nm colloidal gold (AURION, supplied at an OD_520nm_ of 1.0) or haloferritin (GE Healthcare), applied to Quantifoil R2/2 grids and vitrified in liquid ethane using a home-made plunge-freezing device. Images were obtained on an FEI Tecnai F30 Polara transmission electron microscope operating at 300 kV acceleration voltage equipped with a post-column energy filter (GIF Tridiem 863, Gatan) and 2k × 2k CCD camera, or a K2 Summit direct electron detector. Tilt series were collected between ± 60°, with a step size of 2° using the FEI automatic tomogram collection software package or Gatan Digital Micrograph. All images were collected energy filtered (slit size 20 eV) in a defocus range of 2 to 7 μm. Magnifications of 61,000 x resulting in a pixel size at the specimen level of 3.56 Å on the CCD or 3.49 Å on the K2 were used. Tomographic volumes were reconstructed with IMOD and the contrast was enhanced using non-linear anisotropic diffusion (78). Segmentation was performed manually with the program AMIRA (Mercury Systems).

To average the S-layers, tomograms binned twofold and non-linear anisotropic diffusion -filtered (78) were used. Cylindrical models were drawn overlapping with the chosen S-layer cylinders in the cryotomograms. Particle vectors were picked on the cylinders by applying a random oblique grid. Subvolumes were extracted, aligned and averaged using PEET (79, 80). Duplicate removal was conducted to prevent overfitting. A total of 816 (with CaCl_2_) and 1182 (without CaCl_2_) subvolumes were used with dimensions of 80x80x80 pixels. The resolution of the averages in the presence and absence of CaCl_2_ was determined at 31 Å, or 35 Å respectively using Fourier Shell Correlation at the 0.5 criterion. The averages were visualized and segmented using UCSF Chimera (81). Using the IMOD command cloneModel, the average volumes were then plotted onto the original tomograms to obtain 3D models of the reassembled S-layers.

### SymProFold predictions

The SbsC S-layer assembly was generated using a modified protocol of the SymProFold (38) framework. After segmenting the SbsC sequence into the nine structural domains, a set of subchains was generated, each spanning a distinct consecutive series of domains. From this set, combinations of subchains were modelled as oligomeric complexes using AlphaFold 3 (82). Partial models were iteratively superposed, and the growing assembly was analyzed for potential symmetry operations. Upon identification of a symmetry group, the contents of the corresponding primitive unit cell and its lattice parameters were determined. These parameters were then used to generate an 3 × 3 layer fragment of the lattice. The assembled models were subsequently evaluated using validation metrics, including steric clashes, deviations from the lattice plane and symmetry-equivalent positions, and prediction model confidence scores.

### Sequence alignment

Sequence alignment of the full length, domain 4, and domain 7 sequences of Sap and EA1 from *B.* *anthracis* (P49051, P94217), *B. thuringiensis* (A0A1C4APM1, A0A1C4APN4), *B.* *cereus* (A0A150B2Y3, B7HXP5), and SbsB (Q45664), SbsC (O68840) from *G.* *stearothermophilus* was performed with Clustal Omega (83).

### Visualization

All structure representations in the main paper were produced with UCSF ChimeraX (84) Supplementary Figures were also prepared in PyMOL (http://www.pymol.org/; Schrodinger LLC), sequence alignment results (percent identity matrix) were plotted with matplotlib (85) and seaborn (86). Final adjustments and arrangement were done in Inkscape.

### CD spectroscopy

Circular dichroism measurements were performed using a Jasco J-715 spectropolarimeter and a thermostated quartz cuvette with a cell where the path length is 0.02 cm. Samples were dialyzed overnight against 0.1 M Tris-acetate pH 7.4 and 1 mM calcium acetate. On the following day, half of each protein solution was removed and dialysis continued with the 0.1 M Tris-acetate pH 7.4 buffer containing 1 mM EDTA. Protein concentrations were in the range 0.5 to 1.1 mg/ml and were determined by NanoDrop (average of three measurements).

Temperature induced unfolding (25–95 °C) and refolding (95–25 °C) of the protein fragment was followed by recording of the CD signal every 5 °C at a pre-set wavelength. The temperature scan for rSbsC_(541-759)_ was recorded at a wavelength of 200 nm and for rSbsC_(541-882)_ and rSbsC_(642-882)_ at 222 nm. Thermal denaturation figures and T_m_ values were calculated using Origin 5 (Microcal, GE Healthcare). Spectra and thermal denaturation curves are shown in Fig. S3. The melting temperatures are summarized in Table S2.

### 2D crystallization and electron crystallography

The protein fragments rSbsC_(31-1099)_, rSbsC_(31-820)_ and rSbsC_(258-1099)_ were prepared as previously reported (24). Also an adapted protocol was established for the full length protein, that included additional purification step to ensure high purity. In short, *E. coli* BL21(DE3) cells harboring the respective pET3a-construct were grown overnight at 30 °C in LB medium supplemented with ampicillin to a final concentration of 100 mg/L. The main culture (0.5 L LB with 100 mg/L ampicillin in 2 L baffled flasks) was inoculated 1:100 with the overnight culture, grown to an OD_600_ between 0.6 to 0.9 and induced with 0.5 mM IPTG. Expression was performed overnight at 30 °C and 230 rpm shaking. Cells were harvested by centrifugation at 16.1 krcf, 4 °C, for 10 min and stored at −20 °C until cell lysis was performed. For lysis, cell pellets were thawed on ice and resuspended in 50 ml cell lysis buffer (20 mM Hepes/NaOH pH 8.0, 100 mM NaCl). Cells were lysed by sonication (Bandelin Sonopuls, 1 min total sonication time, 50% duty cycle and output control 5) on ice. Afterwards the samples were centrifuged for 10 min, 4 °C and 16.1 krcf and the supernatants were transferred into new safe lock tubes. The pellets containing SbsC proteins were resuspended in 5 ml of the buffer containing 2M GdHCl and 50 mM Tris/HCl pH 7.2 and applied onto a Superdex 200 column (XK26, 51 cm), using the same GdHCl-containing buffer for elution. Fractions containing S-layer proteins, as identified by SDS-PAGE, were pooled and dialyzed against distilled water for at least 24 h at 4 °C and lyophilized for storage. For obtaining 2D crystals, proteins were dissolved in 5 M guanidinium-HCl and dialyzed in dialysis buttons (20–100 μl) against different buffer/salt solutions (Table S3). For microscopy, 2 μl of dialyzed solution containing 2D crystals was pipetted onto a 400-mesh copper electron microscope grid coated with a continuous carbon film. The sample was negatively stained with 2 μl of 2% ammonium molybdate for 15 s and the grid was blotted with Whatman No. 1 paper and air dried. Micrographs were recorded on a Philips CM120 transmission electron microscope operating at 120 kV acceleration voltage in low dose mode (Fig. S4). For all three samples non-tilted images were collected at defocus values of 250 to 700 nm. Images of rSbsC_(31-820)_ and rSbsC_(258-1099)_ used for projection map calculation were recorded on a 2k × 2k CCD camera (Gatan Inc.) at a nominal magnification of 45000 x or 59000 x. Individual images were processed with 2dx_image (87). Projection maps of non-tilted images were calculated using 2dx_merge (88). Observations with IQ = 1 to 7 were used.

## Data availability

All atomic model coordinates have been deposited in the RCSB, respectively. PDB accession numbers are: 4UJ6, 4UID, 4UJ7, 4UJ8, 5FTY, 5FTX, 4UIE, 4UIC. All software packages used in this study are publicly available. Any additional information required to reanalyze the data reported in this article is available from the lead contact upon request.

## Supporting information

This article contains supporting information (26, 40, 41, 42).

## Conflict of interest

The authors declare that they have no conflicts of interest with the contents of this article.
