# Supplementary material for: Self-assembly of surface layer protein SbsC from Geobacillus stearothermophilus and common principles of Bacilli S-layers
Source: J Biol Chem. 2026 Aug 24;302(10):113461. doi: 10.1016/j.jbc.2026.113461 (PMC13635256; doi:10.1016/j.jbc.2026.113461)
Supplement: Supplementary file 1 [file mmc1.docx]

**Supporting Information to:**

Self-assembly of surface layer protein SbsC from *Geobacillus stearothermophilus* and common principles of Bacilli S-layers

Christoph Grininger^1*^, Lavinia Gambelli^2,3^, Anđela Đordić^1^, Markus Eder^1^, Christoph Bulhheller^1^, Theo Sagmeister^1^, Gustav Oberdorfer^4,5^, Janet Vonck^6^, Uwe B. Sleytr^7^, Walter Keller^1,5,8^, Bertram Daum^2^, Tea Pavkov-Keller^1,5,8*^

1 Institute of Molecular Biosciences, NAWI Graz, University of Graz, 8010 Graz, Austria

2 Living Systems Institute, University of Exeter, EX4 4QD Exeter, UK

3 Library Services Research Support, University of Bristol, BS1 5DS Bristol, UK

4 Institute of Biochemistry, University of Technology, 8010 Graz, Austria

5 BioTechMed-Graz, 8010 Graz, Austria

6 Max Planck Institute of Biophysics, Department of Structural Biology, 60438 Frankfurt am Main, Germany

7 Institute of Synthetic Bioarchitectures, University of Natural Resources and Life Sciences Vienna; 1190 Vienna, Austria

8 BioHealth, Field of Excellence BioHealth – University of Graz, 8010 Graz, Austria

^*^ corresponding authors: [christoph.grininger@uni-graz.at](mailto:christoph.grininger@uni-graz.at); [tea.pavkov@uni-graz.at](mailto:tea.pavkov@uni-graz.at)

**Contents**

[Supplementary Figures	S2](#_Toc236302774)

[Supplementary Tables	S11](#_Toc236302775)

# Supplementary Figures

**Figure S1. Schematic representation of all SbsC fragments for which crystal structures are reported in this work.** The lines underneath the colored and numbered domain layout span the included residue ranges of the given construct stated on the left side.

**Figure S2. Representation of the four Ca^2+^ coordination sites (PDB: 4UJ8).** **A**, **B** and **C** – calcium binding sites in domain 6 and **D** – Ca^2+^ binding site in domain 7. Domain 6 is colored in olive green and domain 7 in pink. Calcium ions are shown as orange spheres, water molecules as blue spheres and coordinating residues are in stick representation.

**Figure S3. Thermal denaturation of rSbsC fragments in presence and absence of Ca^2+^.** CD spectra upon heating in 20 mM Tris-acetate buffer (pH 7.4) with 1 mM EDTA (A) and in the same buffer with 1 mM Ca^2+^ (B). Thermal denaturation upon heating 25-95 °C is shown in C.

**Figure S4. TEM analysis of typical rSbsC_(31-1099)_, rSbsC_(31-920)_ and rSbsC_(258-1099)_ 2D crystals.** (A) For rSbsC_(31-1099)_ cylinders, as well as some double-layered sheets could be observed. A representative Fourier transform is shown. (B) Cylinders and some sheets were observed for rSbsC_(31-920)_. (C) rSbsC_(258-1099)_ crystallized as cylinders. Samples were negatively stained with 2% ammonium molybdate. (D) EM projection maps calculated for the full-length SbsC protein (center) and its N- and C- terminal truncation fragments (left and right, respectively). All crystals show P1 symmetry with unit cell dimensions of a=108 Å, b=72 Å and γ=82°.

**Figure S5. Validation of AF3 model of full length SbsC.** All available fragment crystal structures of SbsC were aligned to the full length SbsC AF3 model with ChimeraX matchmaker showing local agreement and overall plausibility of the prediction. Prediction model shown in wheat, crystal structures colored by domains as shown in Figure 1.

**Figure S6. Stick representation of the residues involved in the intramolecular inter-domain contacts as observed in rSbsC(447-1099) (PDB 5ftx).** Domain 4 - green; domain 5 - fluorescent green; domain 6 - olive green; domain 7 - pink. A and B: interaction site between domains 5 and 6. C: Stabilization of the linker region between domains 5 and 6. D: Interaction site between domains 4 and 7. Calcium atoms are shown as orange spheres.

**Figure S7. Alignment of the domain pairs from different SbsC fragments.** All indicated structures (PDB codes) are respectively aligned to the N-terminal domain of the structure colored in wheat. Different conformations are seen for the linker between domain 1-2, 2-3, 4-5 and 6-7. Domain coloring follows the scheme shown in Figure 1 and domain numbers are given in colored circles. 2RA1: rSbsC_(31-443)_, 4UJ6: rSbsC_(31-790)_, 4UIC: rSbsC_(31-844)_, 4UID: rSbsC_(443-650)_, 5FTX: rSbsC_(447-1099)_, 4UJ7: rSbsC_(541-759)_, 4UJ8: rSbsC_(642-882)_, 5FTY: rSbsC_(642-882)_

**Figure S8. Electrostatic potential of the assembled SbsC layer.** ESP was calculated with ChimeraX, Left side shows the top-down view onto the environment exposed side of the S-layer, middle shows the side view, right side the surface facing the bacterial cell.

**Figure S9. Comparison of S-layer protein domains from SbsC, SbsB, EA1 and SAP.** Individual domains were aligned to the corresponding domain of SbsC with ChimeraX matchmaker and PyMOL super (representations from matchmaker in ChimeraX). For comparison both RMSD values and aligned amino acids are stated for both methods for each domain. PyMOL super performs structural alignment without prior sequence alignment where matchmaker only structurally aligns residues identified in a sequence alignment.

# Supplementary Tables

**Table S1. SbsC fragments and methodology used for structural characterization.** The list of all determined crystal structures of SbsC fragments from this work, the fragment rSbsC_(31-443)_ is already reported (26).

| **Construct** | **PDB** | **Domains** | **Crystallization** | **Data collection** | **Structure solution method** |
| --- | --- | --- | --- | --- | --- |
| rSbsC_(31-443)_ | 2RA1 | 1-3 | Pavkov et al, 2008 (26) | Pavkov et al, 2008 (26) | MR  Pavkov et al, 2008 (26) |
| rSbsC_(31-790)_ | 4UJ6 | 1-6 | Dordic et al, 2012 (40) | Dordic et al, 2012 (40) | MR  This work |
| rSbsC_(31-844)_ | 4UIC | 1-6 | Pavkov et al, 2003 (42) | Pavkov et al, 2003 (42) | MIRAS  This work |
| rSbsC_(443-650)_ | 4UID | 4-5 | Dordic et al, 2012 (40) | Dordic et al, 2012 (40) | MR  This work |
| rSbsC_(447-1099)_ | 5FTX | 4-9 | this work  Dordic et al, 2012 (40) | this work | MR  This work |
| rSbsC_(541-759)_ | 4UJ7 | 5-6 | Dordic et al, 2012 (40) | Dordic et al, 2012 (40) | MR  This work |
| rSbsC_(642-882)_ | 5FTY 4UJ8 | 6-7 | this work | this work | MR  This work |
| rSbsC_(755-1099)_ | 4UIE | 7-9 | this work  Kroutil et al, 2009 (41) | this work  Kroutil et al, 2009 (41) | MIRAS  this work |

**Table S2. Thermal denaturation of rSbsC fragments in presence and absence of Ca^2+^.** Overview of T_m_ values for SbsC fragments obtained from CD measurements. For rSbsC_(541-759)_ two transitions are visible as displayed in **Fig. S3C,** both melting temperatures are reported.

| **Construct** | **rSbsC_(541-759)_** | **rSbsC_(642-882)_** | **rSbsC_(541-882)_** |
| --- | --- | --- | --- |
| **Domains** | **5-6** | **5-7** | **6-7** |
| No. of domains | 2 | 3 | 2 |
| No. of Ca^2+^ | 3 | 4 | 4 |
| **T_m_ (+ Ca^2+^) [°C]** | **87.7** | **> 95** | **90-95** |
| **T_m_ (- Ca^2+^) [°C]** | **58/87.9** | **78.9** | **80.2** |
| λ [nm] | 200 | 222 | 222 |

**Table S3. Tested conditions for 2D crystallization.**

| **Parameter** | **Tested range** | **Best condition** |
| --- | --- | --- |
| pH | 10 mM, 4-9 | 7 (HEPES) |
| Protein concentration | 0.1-8 g/l | Lower concentrations |
| Salt | NaCl, LiCl, CaCl_2_, ZnCl_2_ | ZnCl_2_ |
| Salt concentration | 0-1 M | 10-100 mM |
| Dialysis time | 10 min-overnight | 2-4 h |
| Ligand | +/- SCWP | No change |
| Protein:ligand-ratio | 2:1, 1:1, 1:2, 1:4 | No change |
| Stain | AM, UA, PT | AM (pH 7) |
| Temperature | 4, 22, 55, 60 °C | 22 °C |

**Table S4. Data collection and processing statistics** for additional crystal forms and Au-heavy atom derivative of rSbsC_(755-1099)_ used for structure solution (MIRAS and multicrystal averaging).

|  | **rSbsC_(755-1099)_ #27, form2** | **rSbsC_(755-1099)_ + Au #27** | **rSbsC_(755-1099)_ #93** |
| --- | --- | --- | --- |
| *Crystal parameters* |  |  |  |
| Beamline | EMBL/DESY Hamburg, X11 | EMBL/DESY Hamburg, X11 | Elettra Trieste, XRD1 |
| Space group | *P*3_1_21 | *P*4_1_2_1_1 | *P*2_1_2_1_2 |
| Unit-cell parameters (Å, °) | 69.8, 69.8, 196.0  90, 90, 120 | 68.5, 68.5, 313.3  90, 90, 90 | 92.0, 177.8, 52.7  90, 90, 90 |
| *Data collection* |  |  |  |
| Resolution (Å) | 20-3.1 (3.15-3.1) | 20-4.0(4.07-4.0) | 18-4.0 (4.09-4.0) |
| Wavelength (Å) | 0.9486 | 1.0246 | 1.0 |
| *R*_merge_ (%) | 18.5 (46.0) | 15.6 (42.5) | 14.9 (56.4) |
| <*I*/ σ (*I*)> | 7.2 (2.1) | 18.0(4.4) | 10.1 (2.2) |
| No. of unique reflections | 9719 (437) | 6827 (292) | 7965 (514) |
| Redundancy | 4.9 (3.4) | 18.4 (13.7) | 11.0 (6.4) |
| Completeness (%) | 91.0 (83.6) | 98.8 (86.1) | 99.7 (97.5) |
| Wilson B factor | 44 | 44.7 | 84.8 |

Values in parentheses are for the highest resolution shell.

**Table S5. Data collection and processing statistics** for rSbsC_(642-882)_ and rSbsC_(447-1099)_ crystals.

|  | rSbsC_(642-882)_ P1 | rSbsC_(642-882)_ P2_1_ | rSbsC_(447-1099)_ |
| --- | --- | --- | --- |
| *Crystal parameters* |  |  |  |
| Beamline | ESRF, BM30A | ESRF, BM30A | ELETTRA, XRD1 |
| Space group | *P*1 | *P*2_1_ | *C*2 |
| Unit cell parameters [Å,°] | 50.9, 54.5, 92.2  103.6, 105.2, 91.4 | 54.7, 49.0, 91.8  90, 99.9, 90 | 100.6, 48.1, 196.1  90, 96.6, 90 |
| *Data collection* |  |  |  |
| Resolution [Å] | 28.6 – 1.7 (1.8 – 1.7) | 45.2 – 2.5 (2.65 – 2.5) | 42.5 – 3.46 (3.66– 3.46) |
| Wavelength [Å] | 0.98 | 0.98 | 1.14 |
| R_meas_ [%] | 8.1 (40.8) | 7.7 (64.8) | 18.0 (19.4) |
| R_merge_ [%] | 5.7 (28.9) | 6.5 (55.5) | 14.7 (15.1) |
| Total number of observations | 181965 (27344) | 61070 (9685) | 32360 (3220) |
| Total number unique | 93503 (14974) | 16697 (2609) | 11056 (1454) |
| <*I*/σ(*I*)> | 8.5 (2.4) | 15.5 (2.3) | 6.0 (3.6) |
| Completeness [%] | 91.6 (91.0) | 99.0 (97.8) | 87.6 (72.6) |
| Redundancy | 1.9 (1.8) | 3.6 (3.7) | 2.9 (2.2) |
| Wilson B factor [Å^2^] | 25.4 | 54.5 | 47.6 |

Values in parentheses are for the highest resolution shell.

**Table S6. Refinement statistics for all deposited rSbsC constructs.**

|  | rSbsC_(31-790)_ | rSbsC_(443-650)_ | | rSbsC_(541-759)_ | | rSbsC_(642-882)_  P1 | rSbsC_(642-882)_  P2_1_ | rSbsC_(447-1099)_ | rSbsC_(755-1099)_ | rSbsC_(31-844)_ |
| --- | --- | --- | --- | --- | --- | --- | --- | --- | --- | --- |
| *Refinement* |  | |  | |  |  |  |  |  |  |
| Resolution [Å] | 49.3 – 3.4 | 30 – 2.8 | | 48.3 – 1.54 | | 24.5 – 1.8 | 38 – 2.6 | 35 – 4.1 | 20-3.1 | 25-3.0 |
| R [%] | 18.6 | 25.8 | | 18.98 | | 20.5 | 26.8 | 20.3 | 20.7 | 22.5 |
| R_free_ [%] | 25.3 | 29.0 | | 21.67 | | 24.5 | 30.7 | 26.8 | 28.0 | 27.2 |
| *R.m.s.d. stereochemistry* |  | |  | |  |  |  |  |  |  |
| Bond lengths [Å] | 0.006 | 0.006 | | 0.008 | | 0.007 | 0.003 | 0.007 | 0.006 | 0.005 |
| Bond angles [°] | 1.001 | 1.012 | | 1.242 | | 1.07 | 0.760 | 0.791 | 1.012 | 0.920 |
| No. of protein atoms | 5360 | 2798 | | 1594 | | 6958 | 3387 | 4710 | 2798 | 5394 |
| No. of water molecules | 65 | 59 | | 307 | | 742 | 16 | 0 | 59 | 34 |
| Average B factor [Å^2^] | 82.5 | 24.8 | | 21.3 | | 16.12 | 46.34 | 87.26 | 24.8 | 71.5 |
| *Ramachandran analysis* |  | |  | |  |  |  |  |  |  |
| Favoured [%] | 95.3 | 97.0 | | 97.4 | | 96.89 | 95.1 | 88.14 | 97.1 | 95.6 |
| Allowed [%] | 4.6 | 3.0 | | 2.6 | | 3.11 | 4.9 | 9.86 | 2.9 | 4.3 |
| Disallowed [%] | 0.1 | 0 | | 0 | | 0 | 0 | 2.00 | 0 | 0.1 |
| PDB code | 4uj6 | 4uid | | 4uj7 | | 4uj8 | 5fty | 5ftx | 4uie | 4uic |
